# A hybrid computational model of cancer spheroid growth with ribose-induced collagen stiffening

**DOI:** 10.3389/fbioe.2025.1515962

**Published:** 2025-04-09

**Authors:** Margherita Botticelli, John Metzcar, Thomas Phillips, Susan Cox, Pradeep Keshavanarayana, Fabian Spill

**Affiliations:** ^1^ School of Mathematics, College of Engineering and Physical Sciences, University of Birmingham, Birmingham, United Kingdom; ^2^ Intelligent Systems Engineering, Indiana University, Bloomington, IN, United States; ^3^ Randall Centre for Cell and Molecular Biophysics, King’s College London, London, United Kingdom; ^4^ Centre for Computational Medicine, University College London, London, United Kingdom

**Keywords:** mathematical oncology, agent-based model, collective migration, extracellular matrix, ECM stiffness, cancer cell biology

## Abstract

Metastasis, the leading cause of death in cancer patients, arises when cancer cells disseminate from a primary solid tumour to distant organs. Growth and invasion of the solid tumour often involve collective cell migration, which is profoundly influenced by cell-cell interactions and the extracellular matrix (ECM). The ECM’s biochemical composition and mechanical properties, such as stiffness, regulate cancer cell behaviour and migration dynamics. Mathematical modelling serves as a pivotal tool for studying and predicting these complex dynamics, with hybrid discrete-continuous models offering a powerful approach by combining agent-based representations of cells with continuum descriptions of the surrounding microenvironment. In this study, we investigate the impact of ECM stiffness, modulated via ribose-induced collagen cross-linking, on cancer spheroid growth and invasion. We employed a hybrid discrete-continuous model implemented in PhysiCell to simulate spheroid dynamics, successfully replicating three-dimensional *in vitro* experiments. The model incorporates detailed representations of cell-cell and cell-ECM interactions, ECM remodelling, and cell proliferation. Our simulations align with experimental observations of two breast cancer cell lines, non-invasive MCF7 and invasive HCC 1954, under varying ECM stiffness conditions. The results demonstrate that increased ECM stiffness due to ribose-induced cross-linking inhibits spheroid invasion in invasive cells, whereas non-invasive cells remain largely unaffected. Furthermore, our simulations show that higher ECM degradation by the cells not only enables spheroid growth and invasion but also facilitates the formation of multicellular protrusions. Conversely, increasing the maximum speed that cells can reach due to cell-ECM interactions enhances spheroid growth while promoting single-cell invasion. This hybrid modelling approach enhances our understanding of the interplay between cancer cell migration, proliferation, and ECM mechanical properties, paving the way for future studies incorporating additional ECM characteristics and microenvironmental conditions.

## 1 Introduction

The extracellular matrix (ECM) is a complex network of numerous macromolecules present within all tissues outside the cells. It comprises approximately 300 different proteins, with fibrous proteins such as collagens being the most abundant (Yue, 2014). The ECM’s composition and structure, which vary based on tissue type and location, determine distinct mechanical and biochemical properties, which regulate tissue homeostasis, cell differentiation and growth, and largely influence cell migration (Muncie and Weaver, 2018). The physical and mechanical characteristics of the ECM, including fibre orientation, stiffness, viscoelasticity and porosity, affect the cell’s direction of movement, speed and various modes of migration (single or collective) (Yamada et al., 2022). The ECM plays an important role in regulating cell migration during cancer metastasis, which is the primary cause of death in cancer patients (Yamada and Sixt, 2019). More mesenchymal cancer cell lines typically invade as single cells and this mode of invasion is profoundly impacted by ECM pore and fibre size (Rodríguez-Cruz et al., 2024). In solid tumours, when cancer cells migrate collectively, their movement is directed by both cell-cell interactions and their interaction with the ECM (Janiszewska et al., 2020). When cells interact with the ECM, they sense and respond to mechanical cues from the ECM through mechanotransduction pathways, often mediated by integrin signalling (Kanchanawong and Calderwood, 2023). These cues can trigger intracellular signalling cascades, ultimately impacting cell behaviour (Alert and Trepat, 2020; Muncie and Weaver, 2018). The tumour microenvironment differs significantly from healthy microenvironments and is heavily remodelled by cancer cells and fibroblasts (Winkler et al., 2020). Understanding how cancer cells sense and respond to the ECM and its mechanical and biochemical features is crucial to better understanding cancer cell migration and invasion.

Over the years, researchers have investigated cell-ECM interactions in relation to various mechanical properties of the ECM, including stiffness, a material’s resistance to deformation. Notably, increases in ECM stiffness can happen during tumour progression and affect cancer cell behaviour and migration (Wullkopf et al., 2018; Micalet et al., 2023; Jahin et al., 2023). Furthermore, higher ECM stiffness in breast cancers is usually associated with poor prognosis and drug resistance (Joyce et al., 2018; Jahin et al., 2023). Many studies looking at ECM stiffness employ *in vitro* models that simplify the ECM composition, for example, by using only collagen to represent the ECM, and are performed in two dimensions due to ease of sample generation, their simplicity in analysing the results and easier reproducibility (Micalet et al., 2023). However, there are intrinsic differences between cell cultures in two-dimensional (2D) and three-dimensional (3D) matrices. Cell migration features, such as migration speed, directionality, cell morphology and cytoskeletal organisation are profoundly influenced by the surrounding ECM, while key factors seen *in vivo* such as ECM remodelling inherently require an ECM component (Cavo et al., 2016; Gonçalves and Garcia-Aznar, 2021). For example, cells on 2D matrices present a flatter and more spread-out morphology with large flat protrusions (lamellipodia) mainly localised at the leading edge of the cell. In 3D the cells have a more varied morphology and form protrusions on the whole cell surface, such as pseudopodia and invadopodia, adapting to the surrounding ECM (Caswell and Zech, 2018). Therefore, to faithfully replicate 3D *in vivo* tumour microenvironments, we must focus on 3D *in vitro* ECM models rather than relying solely on 2D models. However, creating 3D *in vitro* ECM models presents challenges due to their complexity, both in sample preparation and data analysis, which explains why researchers often turn to 2D experiments to study mechanical effects of the ECM.

A recent review by Micalet et al. (2023) collected various papers investigating the effects of ECM stiffness in 3D *in vitro* models of epithelial cancer cells. Some studies found that matrices with higher stiffness can enhance cell migration, and promote epithelial-to-mesenchymal transition (EMT), a process associated with increased invasiveness (Wei et al., 2015; Stowers et al., 2017). On the other hand, other studies have found that less stiff matrices can drive more invasive phenotypes in cancer cells and spheroids (Staneva et al., 2018; Berger et al., 2020; Jahin et al., 2023). Thus, the effect of ECM on cancer migration and invasion depends not only on stiffness, but on several factors, including the cancer cell type, the involvement of other cells such as fibroblasts, and the composition and structure of the surrounding microenvironment. Furthermore, ECM stiffness in *in vitro* models can be modulated using different techniques, each affecting cell migration differently. One approach involves increasing the collagen density and therefore the extracellular matrix stiffness. However, this alters the structural properties of the ECM, such as its porosity, and influences cell behaviour, including the formation and number of focal adhesions (cell-ECM adhesion sites), which often alters cell migration (Mason et al., 2013). Another method is to modify the alginate hydrogel density. However, this limits cell migration by preventing chemical remodelling of the ECM, essential for cell invasion (Mason et al., 2013). In other studies, the stiffness of the collagen matrix is modulated using non-enzymatic glycation, induced by sugars such as threose and ribose. This process increases cross-links among collagen fibres, increasing fibre stiffness without modifying the structure of the ECM (Staneva et al., 2018; Jahin et al., 2023). The different approaches used in modelling and measuring cancer cell invasion in 3D lead to contradictory results which are also difficult to compare. It is therefore even harder to understand the mechanisms behind cancer cell invasion and their dependence on ECM stiffness.

Given the complexity, cost, and duration of *in vitro* experiments, computational models have become valuable tools in complementing experimental work by replicating setups, providing insights that help interpret results, and exploring scenarios that are difficult to test experimentally (Metzcar et al., 2019; Crossley et al., 2024). Cancer spheroids, commonly used in 3D *in vitro* models to investigate tumour growth and invasion, frequently display collective invasion behaviour. Various computational approaches are employed to model this collective migration, including continuous, discrete, and hybrid models, all of which have been used to examine cancer invasion and interactions between the cells and the surrounding extracellular matrix. In continuous models, the cells and the ECM are represented as densities, characterising them with partial differential equations to describe how they change in space and time (Nguyen Edalgo and Ford Versypt, 2018; Szymańska et al., 2024). In discrete frameworks, agent-based modelling is often used, where each biological element, such as a cancer cell or ECM fibre, is distinct and the interactions between the agents are defined. For example, Prasanna et al. (2024) used a cellular Potts model-based multiscale computational framework to investigate spatial tumour heterogeneity. Finally, hybrid models combine multiple methods, such as modelling the cells using a discrete model and the substrate using a continuous model. Poonja et al. (2023) built a model that combines an off-lattice agent-based model for the cells with a vector field representation of the ECM fibril structure. In this model, the ECM is characterised by fibril orientation and stiffness, which can inhibit cell proliferation or trigger cell migration when respective stiffness thresholds are exceeded.

Several software tools that utilise agent-based models for studying collective cell migration problems have gained popularity. These modelling platforms have been used to build custom computational models to simulate various multiscale and multicellular problems utilising different mathematical frameworks. Notable examples include Chaste (Mirams et al., 2013), CompuCell3D, which uses a cellular Potts model (Swat et al., 2012), and PhysiCell, which employs an off-lattice centre-based agent-based model (Ghaffarizadeh et al., 2018). PhysiCell is an efficient and extensible open-source software tool, able to simulate large numbers of cells in high throughput. It has a growing user community and has been previously used to model the extracellular matrix. Gonçalves and Garcia-Aznar (2021) developed a model for spheroid growth with PhyiCell by defining the ECM as part of the chemical microenvironment with zero diffusivity. The extracellular matrix in PhysiCell has also been modelled as an agent in a PhysiCell addon, named PhysiMeSS (Noël et al., 2024). Here each ECM fibre is represented by cylinders with varying stiffness. Another ECM extension of PhysiCell was developed by Metzcar et al. (2025). They modelled the ECM as a continuum, then discretised into smaller volumetric elements which store information about the ECM fibre orientation, average anisotropy and fibre density.

We present a hybrid discrete-continuous model built upon the ECM framework developed by Metzcar et al. (2025) in PhysiCell, utilising its ECM fibre density feature. The model explores the influence of cell-cell and cell-matrix interactions on cancer spheroid growth at different levels of ribose-induced ECM stiffness. Our model accounts for cell-cell and cell-ECM adhesion and repulsion, ECM remodelling, and cell proliferation with associated inhibition of proliferation function, allowing us to successfully replicate the experimental finding of Jahin et al. (2023), which investigated cancer spheroid growth and invasion of non-invasive MCF7 and invasive HCC1954 cells at different ribose-induced stiffnesses of the ECM. Consistent with the *in vitro* experiments, our results indicate that ribose-induced stiffening can significantly reduce ECM remodelling and confine cancer cell movement, inhibiting spheroid growth and invasion. Moreover, this flexible modelling framework is able to incorporate additional ECM characteristics and microenvironmental conditions, such as fibre orientation and nutrient diffusion, to further refine the dynamics of cancer spheroid-ECM interaction in the future.

## 2 Materials and methods

### 2.1 Experimental data

In this paper, we aim to investigate the mechanisms involved in cancer spheroid growth and invasion into the extracellular matrix. Spheroid growth refers to the expansion of the central spheroid mass and its volume change over time as a result of cell proliferation. On the other hand, invasion describes the penetration of single cells or broad multicellular protrusions into the surrounding ECM. We build the model based on the *in vitro* experiments conducted by Jahin et al. (2023) studying the effect of ribose-induced ECM stiffening on cancer spheroid growth and invasion of non-invasive MCF7 and invasive HCC1954 breast cancer cell lines.

In their study, Jahin et al. (2023) formed tumour spheroids of 200 μ m in diameter using the hanging drop method and subsequently embedded them in a collagen matrix with varying ribose concentrations of 0 mM, 50 mM and 200 mM as in Phillips et al. (2023). They used the non-invasive parental MCF7 and invasive parental HCC1954 cells, both human breast carcinoma cell lines with epithelial-like morphology. To model the ECM, they chose collagen I, derived from rat tail tendons, as it is the most abundant protein component in the extracellular matrix surrounding solid tumours, with supplementary fibronectin also included to allow enhanced cell attachment. During collagen hydrogel formation, ribose, a cross-linker used for non-enzymatic glycation to induce gel stiffening in *vitro* models, was added at appropriate concentrations to increase hydrogel stiffness. More cross-linking between collagen fibres increases the ECM stiffness without altering the matrix organisation and the ligand binding sites for cell-ECM adhesion. This allows for the investigation of the effect of stiffness alone on cancer spheroid growth and invasion. The spheroids were imaged by combining Z-slices at 10 μ m intervals, covering the whole spheroid thickness. The images were captured daily over at least 96 h and were used to track the spheroid invasion.

### 2.2 Model

#### 2.2.1 PhysiCell and general framework

PhysiCell is an open-source cross-platform compatible multiscale modelling tool, based in C++ (Ghaffarizadeh et al., 2018). It employs a hybrid discrete-continuum approach, coupling an agent-based model for the cells with a continuum model for the diffusive microenvironment. The agent-based model is off-lattice and centre-based. Each agent, corresponding to a single cell, is modelled as a sphere, with its position defined by its centre. The continuum microenvironment consists of chemical substrates with associated diffusion coefficients, decay rates, sources and sinks, and initial and boundary conditions. PhysiCell is coupled to an efficient multi-substrate diffusion solver called BioFVM (Ghaffarizadeh et al., 2016) to simulate the chemical microenvironment using reaction-diffusion PDEs. PhysiCell uses multiscale modelling, as it has been developed with the aim of modelling problems in cancer biology and tissue engineering, which involve processes occurring at different time scales. The system is updated using pre-defined and user-defined parameters and functions, making this tool flexible and customisable.

We build our model upon the PhysiCell (version 1.12.0) ECM framework developed by Metzcar et al. (2025). The continuum ECM is defined separately from the chemical microenvironment and is discretised into volumetric elements, or voxels. In our model, each voxel stores information about the local ECM density and we did not include any additional diffusible substrate. The model consists of three parts:

• ECM remodelling, corresponding to changes in ECM density due to degradation by the cells (Section 2.2.2);
• Cell movement, as a result of cell-cell and cell-ECM interactions (Section 2.2.3);
• Cell proliferation, with associated inhibition of proliferation function (Section 2.2.4).

We assume that the ribose concentration affects how the cells interact with the ECM, reducing ECM remodelling and slowing cell migration. For ECM remodelling and cell movement processes, we update the system every mechanics time step $\Delta t_{\mathrm{mech}}$ at the default value of 0.1 min, whilst slower cell phenotype processes, *i.e.*, cell proliferation and volume changes, are updated at a slower rate, every phenotype time step $\Delta t_{\mathrm{cell}}$ at the default value of 6 min (Ghaffarizadeh et al., 2018).

**FIGURE 1:**
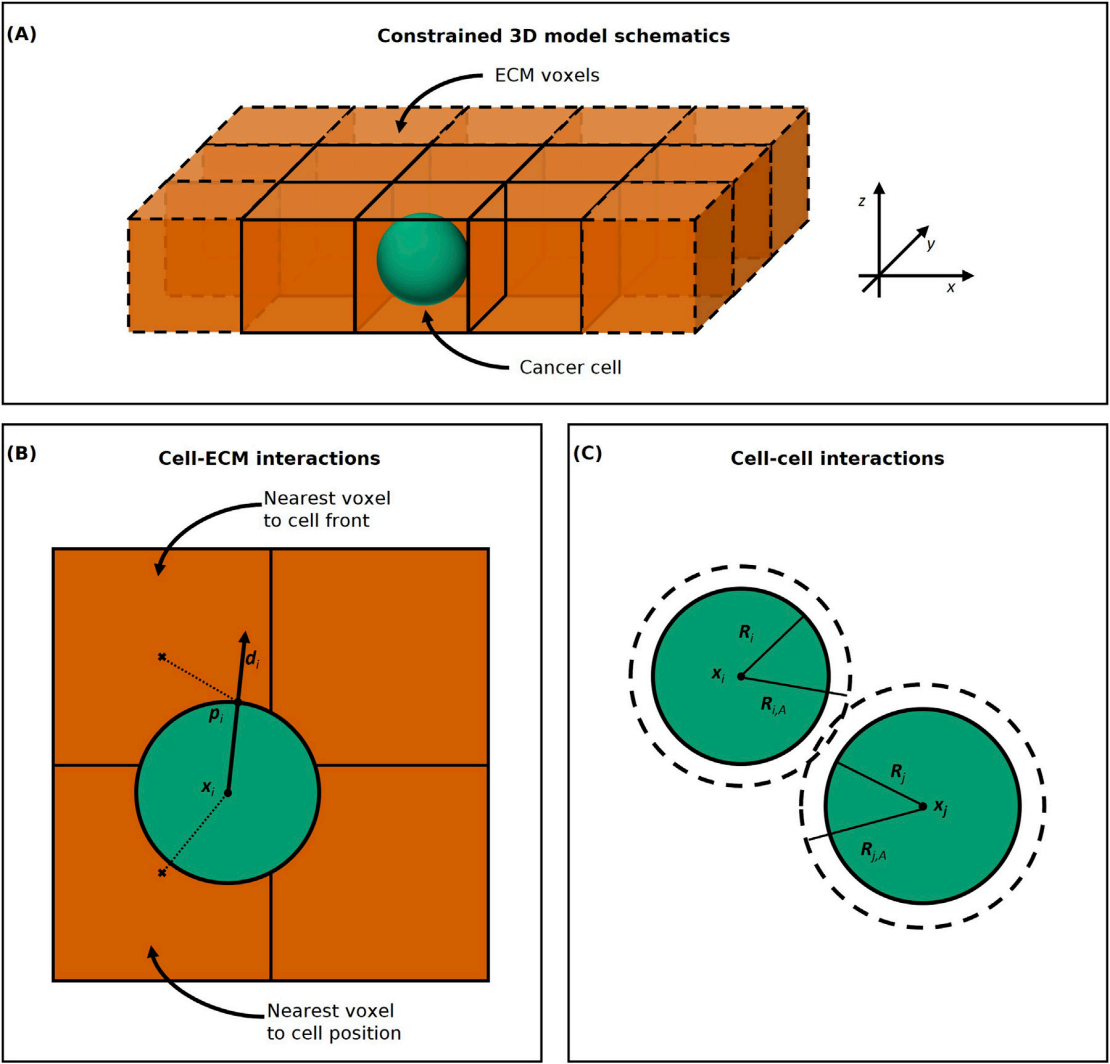
**(A)** Constrained 3D model schematics. The ECM voxels are represented as orange cubes and the cancer cell as a green sphere. The cancer cell agent moves freely within the ECM voxels in the x− and y -directions, but not in the z -direction. **(B)** Cell-ECM interactions computation. The cell (green) interacts with the ECM voxel (orange) whose centre (×) is nearest to either its position $x_{i}$ or its front $p_{i}$ , determined as the point on the cell surface in its direction of movement $d_{i}$ **(C)** Cell-cell interactions computation. Cell $C_{i}$ on the left has a radius $R_{i}$ and an interaction radius $R_{i,A}$ and cell $C_{j}$ on the right has a radius $R_{j}$ and an interaction radius $R_{j,A}$ . The two cells interact when their distance is less than $R_{i,A}+R_{j,A}$ .

We present a pseudo-2D model representing a z -slice image of the experimental data (Figure 1A). The cells are spherical agents with a maximum volume V and corresponding radius R that can interact with the ECM voxels. We use a single layer of ECM voxels inside which the cell agents can move in the x- and y-directions, though their movement is restricted in the z -direction, making it a constrained 3D model (pseudo-2D). This framework was chosen to facilitate the comparison between the simulated and experimental data, as the experimental images were captured from z-slices of the spheroid.

To compute cell-ECM interactions for cell movement and ECM remodelling, we must choose which ECM voxel each cell agent interacts with. In PhysiCell the default voxel accessed for cell-substrate interactions is the nearest voxel (centre) to the position $x_{i}$ of the cell $C_{i}$ (Figure 1B). However, invading cancer cells can form outward protrusions, such as filopodia and invadopodia, to adhere and remodel the ECM fibres mechanically and chemically (Capuana et al., 2020). During chemical remodelling, cancer cells can use these protrusions to secrete soluble or membrane-bound matrix metalloproteinases (MMPs), a class of matrix-degrading enzymes crucial for invasion (Itoh, 2015). For this reason, we introduce an alternative method of selecting the nearest voxel to the cell. When remodelling the ECM, we modify the density of the nearest ECM element to the cell front, corresponding to the nearest voxel to the point $p_{i}$ on the cell surface in its direction of movement $d_{i}$ , as shown in Figure 1B. We also use the nearest voxel to the cell front to find the local ECM density when computing the cell speed due to cell-ECM adhesion (Equation 4). If the cell is not moving, and so $d_{i}=0$ , we select the nearest voxel to the cell position $x_{i}$ (Figure 1B).

For interactions between cell agents, such as cell-cell adhesion, as in PhysiCell, we consider the set of neighbouring cells $N_{i}$ defined as all the cells within interaction distance $R_{i,A}+R_{j,A}$ from the cell $C_{i}$ ’s centre $x_{i}$ (Figure 1C). $R_{i,A}$ and $R_{j,A}$ are the maximum interaction (or adhesion) radii of the cells $C_{i}$ and $C_{j}$ respectively and they are fixed multiples of the cells radii (Ghaffarizadeh et al., 2018).

#### 2.2.2 ECM remodelling

When a cell enters into contact with an ECM element, it remodels the matrix substrate by changing its density ρ∈ [0,1]. We assume the cells degrade the ECM by dynamically reducing its density towards zero. The ECM density update equation is the following:

$$\frac{d\rho }{dt}=-r_{\mathrm{deg,rib}}\rho ,$$ (1)

where $r_{\mathrm{deg,rib}}$ is the cell’s characteristic rate of degradation of the ECM, which depends on the ribose concentration. It has been observed that an increase in ribose and fibre cross-linking through glycation correlates to less ECM remodelling and degradation of the ECM fibres (Francis-Sedlak et al., 2010; Chang et al., 2020). Therefore, we assume that the ECM degradation rate $r_{\mathrm{deg,rib}}$ depends on the ribose concentration rib as follows:

$$r_{\mathrm{deg,rib}}=r_{\mathrm{deg,0}}e^{-\delta rib},$$ (2)

where δ≥ 0 is a parameter that determines how strongly the ribose affects the cell’s base degradation rate $r_{\mathrm{deg,0}}$ , *i.e.*, when the ribose concentration is zero. By choosing δ= 0 we assume that the ribose concentration does not affect the cell’s degradation rate. If δ> 0, as the ribose concentration rib increases, the degradation rate $r_{\mathrm{deg,rib}}$ decreases, tending to zero as rib goes to infinity.

#### 2.2.3 Cell movement

The total velocity $v_{i}$ of a cell $C_{i}$ can be written as

$$v_{i}=v_{i,cc}+v_{i,cm},$$ (3)

where $v_{i,cc}$ is the cell-cell interaction velocity as a result of cell-cell adhesion and repulsion and $v_{i,cm}$ is the cell-matrix interaction velocity as a result of cell-ECM adhesion and repulsion.

##### 2.2.3.1 Cell-cell interactions

To reproduce cell-cell interactions, we use the built-in functions in PhysiCell for cell-cell adhesion and repulsion, in PhysiCell referred to as cell mechanics (Macklin et al., 2012; Ghaffarizadeh et al., 2018; Metzcar et al., 2025). The cell-cell interaction velocity $v_{i,cc}$ (Equation 3) is a result of cell-cell adhesion and repulsion forces. When the distance between two cell centres $\left|x_{j}-x_{i}\right|$ is less than their interaction distance $R_{i,A}+R_{j,A}$ , cell-cell adhesion is activated and the cell agents start pulling each other (Figure 1). On the other hand, the cell-cell repulsion force is activated when two cells start overlapping, so when the distance between the two cell centres $\left|x_{j}-x_{i}\right|$ is less than the sum of their radii $R_{i}+R_{j}$ (Figure 1). This force is used to reproduce the effect of volume exclusion, and resistance to cell deformation when a cell is pushed by other cells.

##### 2.2.3.2 Cell-matrix interactions

The cell-matrix interaction velocity $v_{i,cm}$ (Equation 3) is a result of cell-ECM adhesion and repulsion

$$v_{i,cm}=v_{i,cmr}+v_{i,cma},$$

where $v_{i,cmr}$ is the cell-ECM repulsion velocity and $v_{i,cma}$ is the cell-ECM adhesion velocity.

The cell-ECM adhesion velocity $\left(v_{i,cma}\right)$ is a result of cell adhesion to the ECM fibres and can be written as

$$v_{i,cma}=s_{i,cma}d_{i,cma},$$

where $s_{i,cma}$ is the cell speed due to cell-ECM adhesion and $d_{i,cma}$ is the cell-ECM adhesion direction. The cell-ECM adhesion direction $d_{i,cma}$ is given by a uniform random unit vector, whilst the speed due to cell-ECM adhesion $s_{i,cma}$ is the magnitude of the velocity due cell-ECM adhesion and depends on the ECM density ρ . A higher density of the ECM corresponds to a higher number of cell-ECM adhesion sites. Therefore, we define $s_{i,cma}$ as a linearly increasing function with respect to the ECM density ρ :

$$s_{i,cma}=4\;S_{\mathrm{rib}}\rho .$$ (4)

$S_{\mathrm{rib}}$ is the maximum cell-ECM interaction speed for a given concentration of ribose rib . The factor 4 ensures that when computing the total cell-ECM interaction speed $\left(s_{i,cm}\right)$ , the maximum equals $S_{\mathrm{rib}}$ (see Equation 7). A higher ribose concentration corresponds to more cross-linking between the collagen fibres. This affects the mechanical remodelling of the fibres, as it makes it harder for the cells to realign the ECM fibres, which is essential to allow direct cell migration and invasion (Provenzano et al., 2006). Since we do not account for fibre alignment and orientation in our model, we assume that higher ribose concentration, and collagen stiffness, relate to slower cell migration. Thus, we further assume that $S_{\mathrm{rib}}$ decreases as the ribose concentration increases as follows

$$S_{\mathrm{rib}}=S_{0}e^{-\sigma rib},$$ (5)

where σ≥ 0 is a parameter that determines how strongly the ribose affects the maximum cell-ECM interaction speed at ribose concentration 0 mM $\left(S_{0}\right)$ . Similarly to the role of δ in Equation 2, by setting σ= 0 we assume that the ribose concentration does not affect the cell maximum speed, while if σ> 0 the cell maximum speed decreases as the ribose concentration rib increases, tending to zero as rib goes to infinity.

Further, in a 3D matrix, the ECM fibres act as an obstacle to cell migration when the matrix is dense. When the ECM density ρ is equal to 1 the cells will be fully repelled by the ECM, which will act as a wall, and when ρ is equal to zero there is no repulsion. Therefore, we define the ECM density-dependent cell-ECM repulsion velocity as

$$v_{i,cmr}=-\left(v_{i,cc}+v_{i,cma}\right)\rho .$$ (6)

Equations 4, 6 indicate that as the ECM density increases, both cell-ECM adhesion and repulsion speeds increase, leading to a non-monotonic resultant total cell-matrix speed $\left(s_{i,cm}\right)$ . Assuming that the cell has no neighbours ( $v_{i,cc}=0$ ), we find that the total cell-ECM interaction speed is given by

$$\begin{array}{ll} v_{i,cm} & =v_{i,cma}+v_{i,cmr}\\ & =v_{i,cma}\left(1-\rho \right)\\ \Rightarrow s_{i,cm} & =4S_{\mathrm{rib}}\rho \left(1-\rho \right), \end{array}$$ (7)

which reaches its maximum at ρ= 0.5, where $s_{i,cm}=S_{\mathrm{rib}}$ .

##### 2.2.3.3 Cell-ECM interaction velocity update

The persistence in cell movement is defined as the mean time a cell maintains its direction of motion (Maiuri et al., 2015). Therefore, we update the cell direction due to cell-ECM interaction $d_{i,cm}$ with probability

$$\text{Prob}\left(\text{change }d_{i,cm}\right)=\frac{\Delta t_{\mathrm{mech}}}{T_{\mathrm{per}}},$$

where $T_{\mathrm{per}}$ is the persistence time (Ghaffarizadeh et al., 2018). Instead, the cell speed due to cell-ECM interaction $s_{i,cm}$ gets updated deterministically every mechanics time step $\Delta t_{\mathrm{mech}}$ . In this way, the cell is able to rapidly react to changes in the ECM density and tune its speed accordingly.

#### 2.2.4 Cell proliferation

For cell proliferation, we use a live cell model from PhysiCell (Ghaffarizadeh et al., 2018). This simple model for proliferation consists of cells dividing in any time interval [t,t+Δt] with probability:

$$\text{Prob}\left(\text{division during }\left[t,t+\Delta t\right]\right)=1-e^{-r_{\mathrm{div}}\Delta t}\approx r_{\mathrm{div}}\Delta t,$$

where $r_{\mathrm{div}}$ is the cell proliferation (or division) rate. When dividing, the cell will halve its volume, duplicate the cell with all its state and parameter values and place the daughter cells side by side with their centres inside the radius of the original cell. The daughter cells then grow in volume until reaching the maximum volume V .

However, compression of the tumour spheroid due to confinement and lack of nutrients can slow or arrest cell proliferation (Delarue et al., 2014; Engin et al., 2017; Ahn et al., 2024). The spheroid can be compressed when the surrounding ECM is too dense and is not degraded quickly enough, slowing proliferation (Delarue et al., 2014). Nutrient diffusion depends on the porosity of the ECM, which in turn depends on the density of the fibres (Ahn et al., 2024). Furthermore, the cells in the centre of the spheroid are less exposed to nutrients, since the cells in the outer layer consume the nutrients first (Pinto et al., 2020). Therefore, as we chose not to include nutrient diffusion in the current model, we simplify inhibition of proliferation by assuming that proliferation is inhibited when the cells are surrounded by neighbours (the number of neighbours above a pre-defined overcrowding threshold) and is slowed down by the presence of extracellular matrix. We rewrite the probability of division in any time interval [t,t+Δt] as

$$\text{Prob}\left(\text{division during }\left[t,t+\Delta t\right]\right)\approx r_{\mathrm{div}}f_{IP}\left(N_{i},\rho \right)\Delta t,$$

where $f_{IP}$ is the inhibition of proliferation function defined as

$$f_{IP}\left(N_{i},\rho \right)=\left\{\begin{array}{cc} 1-\rho \; & \text{if }0<N_{i}<N_{\max}\\ 0\; & \text{if }N_{i}\geq N_{\max} \end{array}\right.$$ (8)

with $N_{i}$ being the number of neighbours of the cell $C_{i}$ , $N_{\max}$ the overcrowding threshold and ρ∈ [0,1] the ECM density of the nearest voxel to the cell position.

### 2.3 Statistical analysis

To compare our results with the experimental data, we calculated spheroid area growth relative to the initial time, cell count and Delaunay mean distance between cells in Python version 3.10.12. Given the stochastic nature of our model, we ran 10 replicates for each simulation and computed the mean and 25th/75th percentile of spheroid area growth relative to the initial time, cell count and Delaunay mean distance every 60 min. Simulations were performed on the University of Birmingham’s high-performance computing (HPC) cluster, BlueBEAR. We utilised a single node ( 2×56 -core Intel^®^ Xeon^®^ Platinum 8570) and ran batches of 20 simulations simultaneously, each allocated 4 GB of RAM. Completion times varied with the number of agents, ranging from approximately 45 s to 5 min, with most simulations finishing within 2–3 min.

**FIGURE 2:**
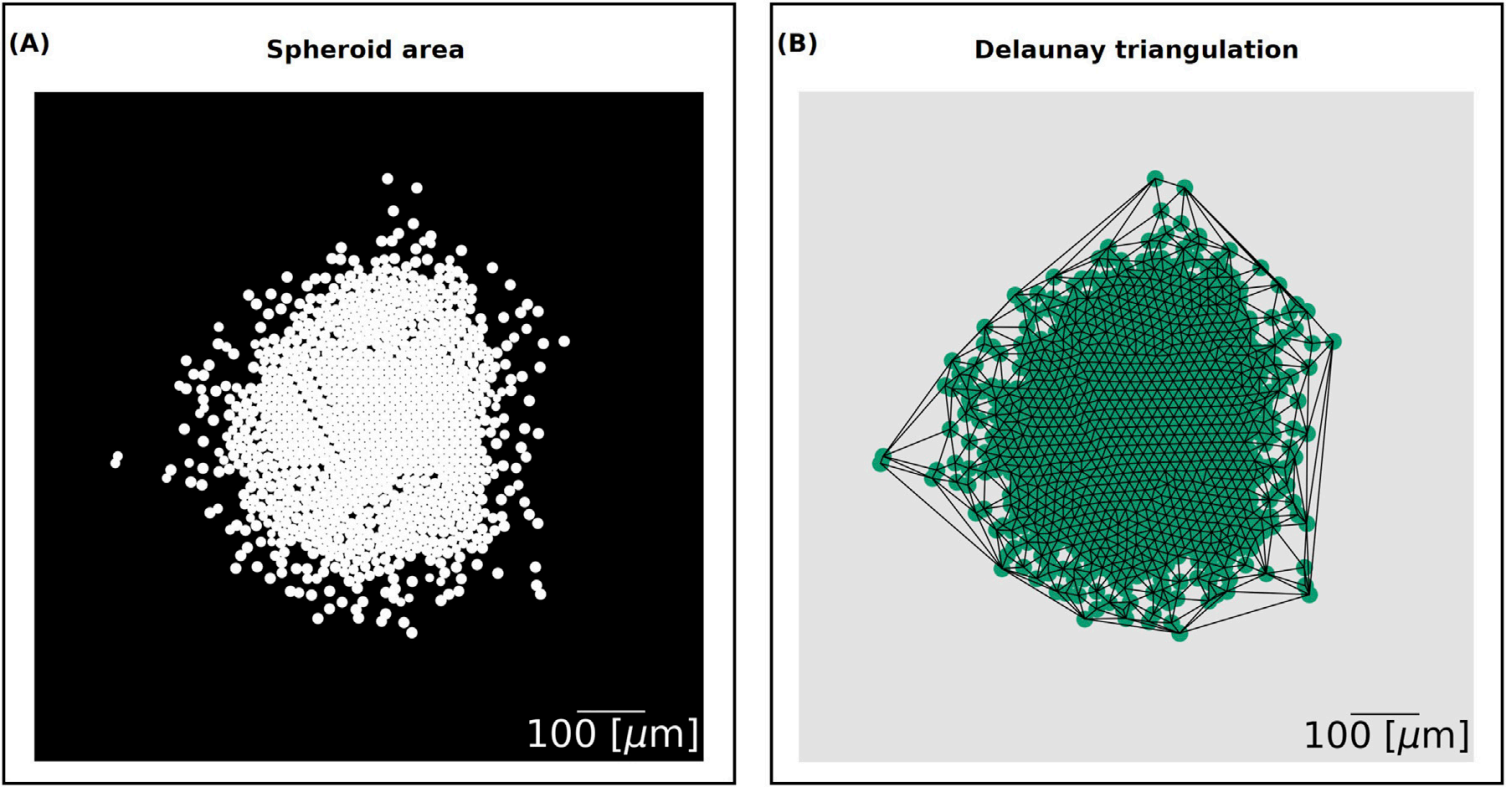
**(A)** Spheroid area output. The domain is divided into a 5000×5000 grid. The grid elements that overlap with a cell have value 1 and are shown in white. The grid elements corresponding to the background have value 0 and are shown in black. The spheroid area is computed by summing together the elements of the grid and rescaling to the original size of the domain. **(B)** Delaunay triangulation output. We generate a network that connects the centres of the cells using the spacial algorithm Delaunay from the Python library SciPy. The scale bar on the bottom right is of length 100 μ m.

We computed the spheroid area growth relative to the initial time by calculating the area covered by the cells at each time point and dividing it by the area covered at the initial time t= 0 min. The spheroid area was approximated by dividing the entire domain into a 5000×5000 , initially setting all grid elements to a value of 0. This baseline value represents unoccupied space. We then drew disks of value 1 at the coordinates of each cell’s centre with their corresponding radius (Figure 2A). Overlaps were ignored, as grid elements covered by multiple cells are only counted once. To calculate the total spheroid area, we summed the grid elements with value 1 and rescaled to the original domain size to obtain the spheroid area in $\mu {\text{m}}^{2}$ . This process is analogous to the method used for computing spheroid invasion relative to the initial time in Jahin et al. (2023).

For the cell count, we tallied the total number of cells in the simulations at each time point, corresponding to the number of nuclei in a slice of the experimental data.

Finally, the Delaunay mean distance measures the proximity of cells. It utilises the Delaunay triangulation, the dual graph of the Voronoi diagram, of a set of points. The edges of the graph form triangles whose circumscribed circles do not contain any other point. We used the cell centres as the input nodes of the network and generated the Delaunay network using the spacial algorithm Delaunay from the Python library SciPy version 1.11.1 (Figure 2B). We then computed the mean edge length between the nodes to find the Delaunay mean distance.

## 3 Results

### 3.1 Impact of key cell-ECM interaction parameters on spheroid growth

Invasion and migration of cancer cells from a cancer spheroid into the surrounding extracellular matrix depends strongly on how they interact with the ECM (Yamada et al., 2022). The cancer cells need to remodel the ECM to enable invasion, and can then use the adhesion sites on the collagen fibres to propel themselves and invade further. In our model, to study such cell-ECM interactions, we consider different biophysical parameters representing cell-ECM cross-talk and specific properties of the ECM. We control how quickly the agent cells reduce locally the ECM density by changing the degradation rate ( $r_{\mathrm{deg,0}}$ , Equation 1). In turn, the ECM density affects the cell’s speed (Equations 4, 6), which reaches its maximum cell-ECM interaction speed $\left(S_{\mathrm{rib}}\right)$ when the ECM density is equal to 0.5. The ECM density also affects the cell’s proliferation rate $\left(r_{\mathrm{div}}\right)$ through the inhibition of proliferation function (Equation 8). In this section, we present the results of our analysis on the degradation rate $\left(r_{\mathrm{deg,0}}\right)$ and maximum cell-ECM interaction speed $\left(S_{0}\right)$ without any ribose at different proliferation rates $\left(r_{\mathrm{div}}\right)$ . Then we analyse the δ and σ parameters, which determine how strongly ribose affects the degradation rate ( $r_{\mathrm{deg,rib}}$ , Equation 2) and the maximum cell-ECM interaction speed ( $S_{\mathrm{rib}}$ , Equation 5) respectively at different ribose concentrations (rib) .

**FIGURE 3:**
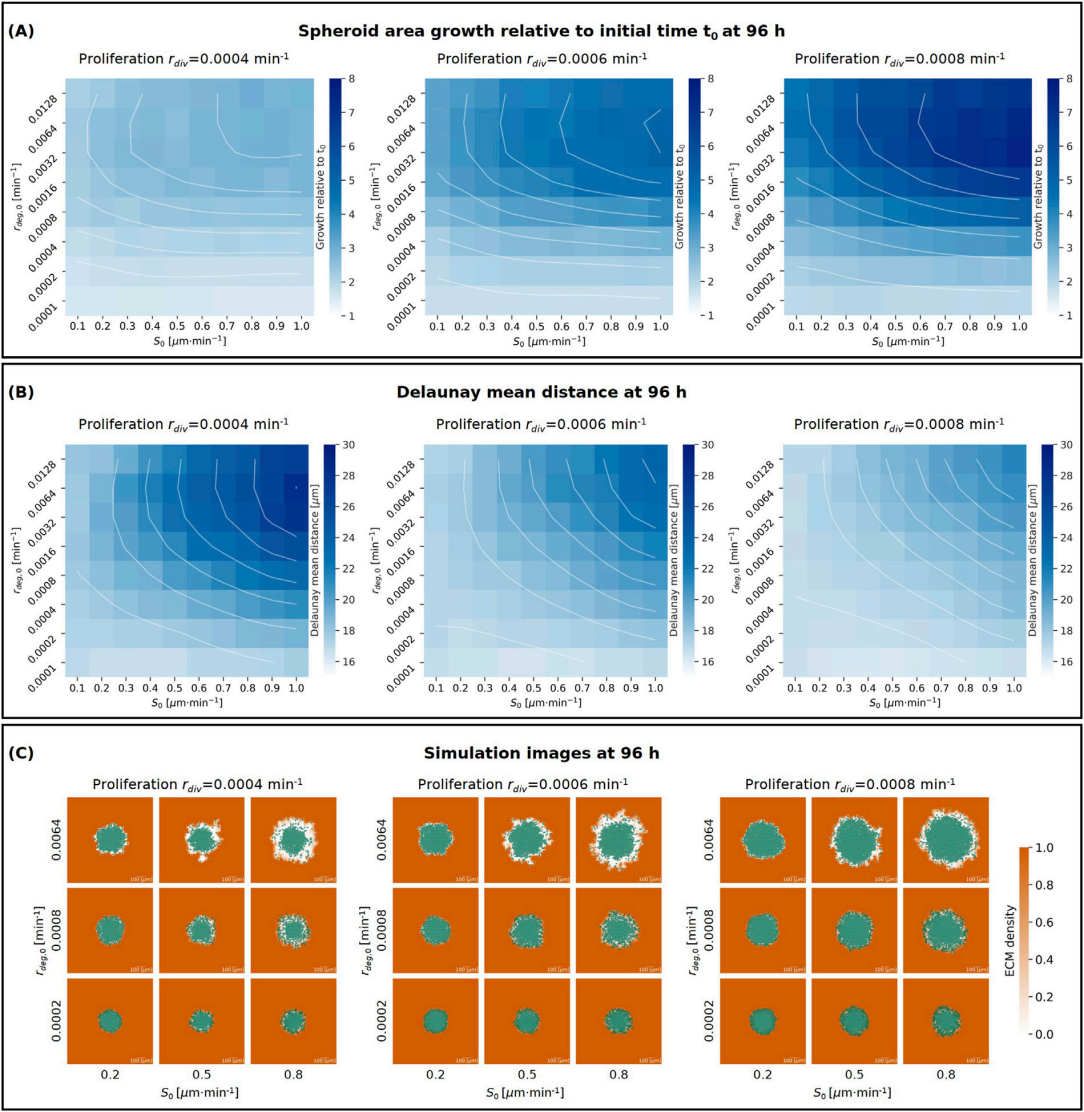
**(A, B)** Heatmaps showing the effects on spheroid area growth relative to the initial time $t_{0}$ **(A)** and Delaunay mean distance **(B)** after 96 h with ribose concentration 0 mM of proliferation rate $r_{\mathrm{div}}$ (columns), maximum cell-ECM interaction speed $S_{0}$ ( x -axis) and degradation rate $r_{\mathrm{deg,0}}$ ( y -axis). The colour intensity represents the mean values over 10 replicates of the spheroid area growth relative to the initial time ranging from 1 to 8 and Delaunay mean distance ranging from 10 μ m to 30 μ m, as shown in the colour bars. Contour lines are also shown in white. Heatmaps with mean values and standard deviations can be found in the Supplementary Figure S1 **(C)** Tables showing the simulation figures at 96 h for varying proliferation rate $r_{\mathrm{div}}$ (columns), maximum cell-ECM interaction speed $S_{0}$ ( x -axis) and degradation rate $r_{\mathrm{deg,0}}$ ( y -axis). The cells are represented in semi-transparent green and the ECM density is in orange taking values between 0 and 1, as shown in the colour bar.

We initiated all simulations with a spheroid of cancer cells of 200 μ m in diameter and homogeneous ECM density with value ρ=1 throughout, except at the spheroid’s location, where the ECM density is zero. We set the overcrowding threshold $N_{max}$ used in the inhibition of proliferation function (Equations 5–8), equivalent to a cell fully surrounded by the other cells (hexagonal packing) (Metzcar et al., 2025). All of the parameters used in the simulations are also listed in the Supplementary Tables S1–S5 and other PhysiCell specific parameters are set to their default values as used in PhysiCell 1.12.0 (Ghaffarizadeh et al., 2018). We examined the impact of the degradation rate $\left(r_{\mathrm{deg,0}}\right)$ and the maximum cell-ECM interaction speed $\left(S_{0}\right)$ on spheroid area growth relative to the initial time and Delaunay mean distance (explained in Section 2.3), holding the ribose concentration at 0 mM. The analysis was conducted for three proliferation rates: $r_{\mathrm{div}}=$ 0.0004 ${\text{min}}^{-1}$ , 0.0006 ${\text{min}}^{-1}$ and 0.0008 ${\text{min}}^{-1}$ (Figures 3A, B).

Our results demonstrate that an increase in proliferation rate $\left(r_{\mathrm{div}}\right)$ enhances spheroid area growth relative to the initial time (Figure 3A) and reduces the Delaunay mean distance (Figure 3B). This is expected, as faster cell division leads to a denser cell population, which is correlated to a lower average cell-cell distance.

Further, increasing the maximum cell-ECM interaction speed $\left(S_{0}\right)$ leads to enhanced spheroid growth and a higher Delaunay mean distance when the degradation rate $r_{\mathrm{deg,0}}$ is above 0.0001 ${\text{min}}^{-1}$ , and to little change when equal to 0.0001 ${\text{min}}^{-1}$ (Figures 3A, B). A higher $S_{0}$ allows more cells to detach from the spheroid, reducing the number of neighbours, and consequently avoiding proliferation arrest. It also enables cells to access and degrade more areas of the ECM, further promoting proliferation. Additionally, with faster cell migration, cells at the spheroid’s edge become more dispersed, contributing to the increase in Delaunay mean distance.

Finally, we observed that increasing the degradation rate $r_{\mathrm{deg,0}}$ does not always induce a monotonic increase in spheroid area growth relative to the initial time and Delaunay mean distance. Generally, increasing the degradation rate enhances spheroid growth (Figure 3A) and increases cell-cell distance, leading to a higher Delaunay mean distance (Figure 3B). This occurs because a higher degradation rate reduces ECM density around the spheroid, which in turn positively affects proliferation and invasion. However, when the degradation rate is excessively high ( $r_{\mathrm{deg,0}}=$ 0.0128 ${\text{min}}^{-1}$ ), the ECM is degraded too quickly, which inhibits migration and invasion. Therefore, for fixed proliferation rate $r_{\mathrm{div}}$ and maximum cell-ECM interaction speed $S_{0}$ , as the degradation rate $r_{\mathrm{deg,0}}$ increases, the spheroid growth slows down (Figure 3A) and the Delaunay mean distance decreases (Figure 3B).

Figure 3C shows simulation images at the final time point (96 h) for $S_{0}=$ 0.2, 0.5 and 0.8 μ m $\cdot {\text{min}}^{-1}$ , $r_{\mathrm{deg,0}}=$ 0.0002, 0.0008 and 0.00064 ${\text{min}}^{-1}$ and $r_{\mathrm{div}}=$ 0.0004, 0.0006 and 0.0008 ${\text{min}}^{-1}$ . These images illustrate that higher $S_{0}$ values lead to greater cell dispersion at the spheroid’s edge. When combined with higher ECM degradation $r_{\mathrm{deg,0}}$ more single cells are observed migrating away from the spheroid. Thus, increased degradation rate and maximum cell-ECM interaction speed contribute to the formation of protrusions in the spheroid. In contrast, lower degradation levels and migration speed limit spheroid growth, resulting in a more rounded spheroid shape.

**FIGURE 4:**
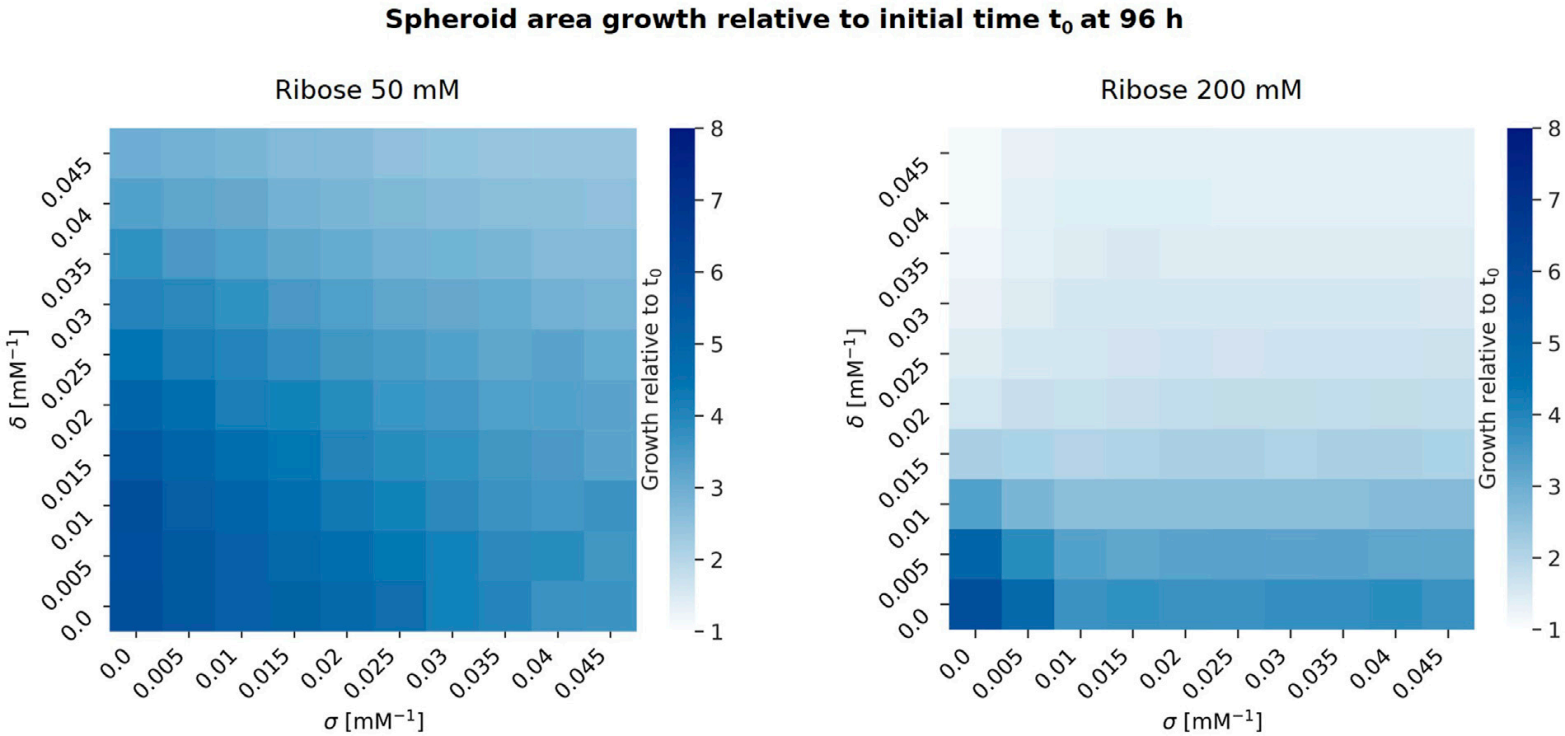
Heatmaps showing spheroid growth relative to the initial time $t_{0}$ for ribose concentration of 50 mM (left) and 200 mM (right) for varying values of the parameters δ ( y -axis) and σ ( x -axis). We set $r_{\mathrm{deg,0}}=$ 0.0032 ${\text{min}}^{-1}$ , $S_{0}=$ 0.7 μ m $\cdot {\text{min}}^{-1}$ and $r_{\mathrm{div}}=$ 0.00072 ${\text{min}}^{-1}$ . The colour intensity represents the mean values over 10 replicates of the spheroid area growth relative to the initial time ranging from 1 to 8, as shown in the colour bars. Heatmaps with mean values and standard deviations can be found in the Supplementary Figure S2.

We then analysed the impact of the parameters δ and σ on spheroid area growth relative to the initial time (Figure 4). For δ,σ= 0 the functions are constant, so $r_{\mathrm{deg,rib}}=r_{\mathrm{deg,0}}$ and $S_{\mathrm{rib}}=S_{0}$ , while for δ,σ> 0 the functions are monotonically decreasing with respect to the ribose concentration rib , so $r_{\mathrm{deg,rib}}<r_{\mathrm{deg,0}}$ and $S_{\mathrm{rib}}<S_{0}$ for ribose rib greater than zero (Equations 2, 5). For this analysis, we fixed the degradation rate at ribose concentration 0 mM ( $r_{\mathrm{deg,0}}=$ 0.0032 ${\text{min}}^{-1}$ ), the maximum cell-ECM interaction speed at ribose concentration 0 mM ( $S_{0}=$ 0.7 μ m $\cdot {\text{min}}^{-1}$ ) and the proliferation rate ( $r_{\mathrm{div}}=$ 0.00072 ${\text{min}}^{-1}$ ). The analysis was conducted for two ribose concentrations: rib= 50 mM and 200 mM.

Figure 4 shows that higher ribose concentrations correspond to a decrease in spheroid growth, and increasing either δ or σ also results in reduced spheroid growth. Notably, at 200 mM ribose, when σ is greater than or equal to 0.015 ${\text{mM}}^{-1}$ , spheroid growth remains unchanged for fixed values of δ . This occurs because for σ= 0.015 ${\text{mM}}^{-1}$ the maximum cell-ECM interaction speed is significantly reduced, $S_{200}=S_{0}e^{-\sigma 200}\approx$ 0.035 μ m $\cdot {\text{min}}^{-1}$ compared to $S_{50}=S_{0}e^{-\sigma 50}\approx$ 0.33 μ m $\cdot {\text{min}}^{-1}$ . As a result, as the cell population becomes denser, proliferation is inhibited throughout the spheroid except at its boundary, where proliferation depends on the surrounding ECM density. Consequently, spheroid growth is determined by the rate at which cells degrade the ECM at the boundary, facilitating increased proliferation in this region.

### 3.2 Model captures inhibition of cancer spheroid growth of non-invasive and invasive breast cancer cells when increasing ribose concentration

To begin with, we replicated experiments from Jahin et al. (2023) that study the effect of ribose concentration on two different cell lines of parental breast cancer cells: MCF7 and HCC 1954. MCF7 cells are a non-invasive cell line, which correlates with weaker cell-ECM interactions (Comşa et al., 2015). On the other hand, HCC1954 cells are a more aggressive and invasive cell line, characterised by enhanced contractility, and therefore stronger interactions with the ECM fibres enabling migration, and further ECM remodelling (de Abreu Pereira et al., 2022; Jahin et al., 2023). The *in vitro* experiments showed that with increasing ribose concentration, and therefore collagen fibre stiffness, the spheroid invasion was inhibited for the invasive HCC1954 cells, while the non-invasive MCF7 cells did not invade for any of the ribose concentrations.

**FIGURE 5:**
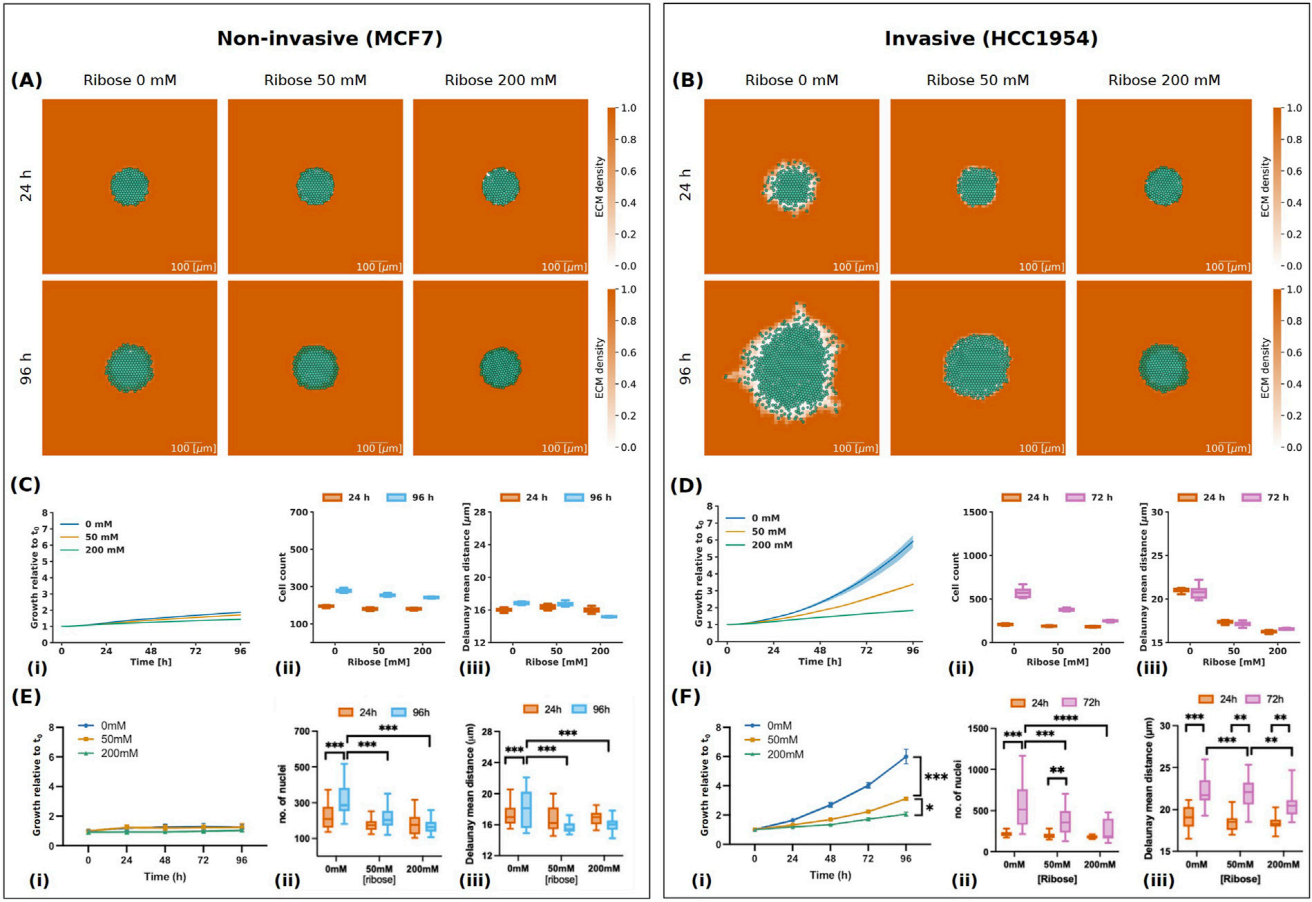
Results of simulations for non-invasive (MCF7) and invasive (HCC 1954) cell lines. **(A, B)** Simulation images of non-invasive **(A)** and invasive **(B)** cell lines respectively, with ribose concentrations of 0 mM, 50 mM and 200 mM at 24 h and 96 h. The cells are represented in semi-transparent green and the ECM density is in orange, with values between 0 and 1 as indicated in the colour bar. The scale bar at the bottom right is 100 μ m in length. Full videos of non-invasive and invasive spheroids with ribose concentration 0 mM can be found in the Supplementary Material. **(C, D)** Simulation results for non-invasive **(C)** and invasive **(D)** cell lines showing line plots of spheroid growth relative to the initial time $t_{0}$ change over time **(i)**, box plots of cell count **(ii**) and box plots of Delaunay mean distance **(iii)**. In the line plots, the ribose concentrations are represented in blue for 0 mM, orange for 50 mM and green for 200 mM. Mean and 25th/75th percentile are shown, with the addition of min/max in box plots. **(E, F)** Adapted with permission from Jahin et al. (2023). Plots for MCF7 (non-invasive) **(E)** and HCC 1954 (invasive) **(F)** cell lines showing growth relative to the initial time $t_{0}$ **(i)**, number of nuclei **(ii)** and Delaunay mean distance **(iii)**. Mean and 25th/75th percentile are shown, with the addition of min/max in box plots. Asterisks indicate statistical significance following ANOVA testing with Sidak’s *post hoc* test (** = p< 0.01, *** = p< 0.001, **** = p< 0.0001).

The cell-ECM interactions in our model depend on two parameters: ECM degradation rate, which controls the ECM remodelling by the cells (Equation 1), and maximum cell-ECM interaction speed, which affects the cell’s movement (Equation 4). Given the different invasiveness of the two cell lines, we assume that the invasive cells have a higher ECM degradation rate and maximum cell-ECM interaction speed than the non-invasive cells. From the wide range of values studied in Section 3.1 (Supplementary Figure S3), we choose the values that lead to simulations matching the experimental observations (Figures 5E, F). Hence, we use the following set of values: ECM degradation rate $r_{\mathrm{deg,0}}=$ 0.0001 ${\text{min}}^{-1}$ and maximum cell-ECM interaction speed $S_{0}=$ 0.1 μ m $\cdot {\text{min}}^{-1}$ for non-invasive cells, and ECM degradation rate $r_{\mathrm{deg,0}}=$ 0.0032 ${\text{min}}^{-1}$ and maximum cell-ECM interaction speed $S_{0}=$ 0.7 μ m $\cdot {\text{min}}^{-1}$ for invasive cells. Further, the strength of the effect of ribose concentration on the cell behaviour depends on the parameters δ for degradation rate $r_{\mathrm{deg,rib}}$ (Equation 2) and σ for maximum cell-ECM interaction speed $S_{\mathrm{rib}}$ (Equation 5). Following Section 3.1 (Figure 4), we use δ= 0.02 ${\text{mM}}^{-1}$ and σ= 0.035 ${\text{mM}}^{-1}$ to match the spheroid growth after 96 h of the invasive cells for ribose concentrations of 50 mM and 200 mM (Figure 5Di). Finally, we set the proliferation rate $r_{\mathrm{div}}=$ 0.00072 ${\text{min}}^{-1}$ , which is the default parameter for the live cell cycle model of MCF10A breast cancer epithelial cells in PhysiCell (Ghaffarizadeh et al., 2018). Following the *in vitro* experiments performed in Jahin et al. (2023), we set the initial spheroid diameter to be 200 μ m (corresponding to 139 cells), with homogeneous ECM density with value ρ= 1 throughout, except at the spheroid’s location, where the ECM density is zero. We study the effect of ribose concentrations, 0 mM, 50 mM and 200 mM, on spheroid area growth relative to the initial time, cell count and Delaunay mean distance, as shown in Figures 5C, D. Simulation images at 24 h and 96 h for non-invasive and invasive cells are shown in Figures 5A, B respectively.

In their experiments with non-invasive MCF7 cells, Jahin et al. (2023) observed that increasing ribose concentration did not affect spheroid growth (Figure 5Ei), consistent with previous reports (Ziegler et al., 2014). Our findings also indicate that spheroid growth was inhibited for the non-invasive cells, with the spheroid area growth at the final time point remaining below 2 for all ribose concentrations (Figure 5Ci). However, while the invasion of MCF7 cells was low, Jahin et al. (2023) observed an increase in the number of nuclei, particularly at 0 mM ribose (Figure 5Eii). Similarly, our simulations showed a larger increase in cell count at 0 mM ribose, with the cell count reaching ∼ 280 after 96 h (Figure 5Cii), which matches the corresponding mean number of nuclei observed *in vitro* (Figure 5Eii). Interestingly, they found that the Delaunay mean distance increased for ribose concentration of 0 mM and it decreased at 50 mM and 200 mM, indicating a denser spheroid for higher ribose concentrations (Figure 5Eiii). In our simulations, we observed that the Delaunay mean distance between 24 h and 96 h slightly increased at ribose concentration 0 mM and decreased for ribose 200 mM, but remained constant at 50 mM (Figure 5Ciii). This means that, in our simulations, the spheroid at 50 mM is less dense than in the experimental data. This can be due to more rapid degradation of the ECM or slower cell proliferation than in the experiments. We also observed a larger decrease in Delaunay mean distance for ribose 200 mM in our simulations compared to the experimental data. This difference could be attributed to the absence of cell death in our *in silico* model, whereas the experimental data show a reduction in the number of nuclei over time likely due to cell death (Figure 5Eii). The higher cell count maintained in the simulations likely results in a denser spheroid and therefore a larger decrease in Delaunay mean distance.

In contrast, the invasive HCC1954 cells exhibited a reduction in spheroid growth and the number of nuclei with a ribose concentration increase *in vitro* (Figures 5Fi, ii). Our simulations closely matched the experimental data, resulting in a spheroid growth of ∼ 6 after 96 h and a cell count of ∼ 570 after 72 h for ribose 0 mM, spheroid growth of ∼ 3.4 after 96 h with a cell count of ∼ 370 after 72 h for ribose 50 mM, and spheroid growth of ∼ 1.8 after 96 h and a cell count of ∼ 250 after 72 h for ribose 200 mM (Figures 5Di, ii). Further, Jahin et al. (2023) found that the Delaunay mean distance at 72 h was higher than that at 24 h, for all ribose concentrations considered. However, it decreased with an increase in the ribose concentration (Figure 5Fiii). Our simulations showed that the Delaunay mean distance at 24 h and 72 h is almost the same for each ribose concentration, but lowers as the ribose increases (Figure 5Diii). However, we see that the Delaunay mean distance does not remain constant over time (Supplementary Figure S4Biii). We noticed that the Delaunay mean distance at ribose 0 mM initially increases and peaks between 24 h and 48 h, before decreasing. While we see an overall increase in Delaunay mean distance for ribose concentration 0 mM, as seen experimentally, our model predicts a rapid increase of Delaunay mean distance and has a similar value at 24 h and 72 h, in contrast to the experiments. With only two experimental time points, we cannot capture the dynamics in the first 24 h and between 24 h and 72 h. Including more time points would allow us to better understand variations over time and a more accurate representation of how the spheroid evolves.

With our choice of parameters, we observe that low degradation rate and maximum cell-ECM interaction speed inhibit both invasion and proliferation of the spheroid. This is mainly due to the cell-ECM repulsive velocity $v_{\mathrm{cmr}}$ (Equation 6) and the inhibition of proliferation function (Equation 8). As observed in the parameter analysis in Section 3.1, low degradation rate and maximum speed make the spheroid denser thanks to the ECM acting as a wall because of the repulsive velocity. Proliferation is inhibited at the centre of the spheroid due to the high number of neighbours and at the edge due to the high ECM density. The chosen values for δ and σ in our simulations give low degradation rates and maximum cell-ECM interaction speeds for the invasive cells at ribose concentrations of 50 mM and 200 mM. The degradation rates at ribose concentrations 50 mM and 200 mM are $r_{\mathrm{deg,50}}\approx$ 0.001 ${\text{min}}^{-1}$ and $r_{\mathrm{deg,200}}\approx$ 0.00006 ${\text{min}}^{-1}$ , while the maximum cell-ECM interaction speeds are $S_{50}\approx$ 0.1 μ m $\cdot {\text{min}}^{-1}$ and $S_{200}\approx$ 0.0006 μ m $\cdot {\text{min}}^{-1}$ . This indicates that both ECM degradation and cell speed are substantially reduced for the invasive cells as the ribose concentration increases, which lowers tumour invasion and proliferation. Thus, our simulations are in line with the observation by Jahin et al. (2023), that ribose-induced cross-linking of collagen possibly reduces ECM remodelling and migration, slowing spheroid growth and invasion.

### 3.3 MMPs inhibition for invasive cells inhibits spheroid area growth

**FIGURE 6:**
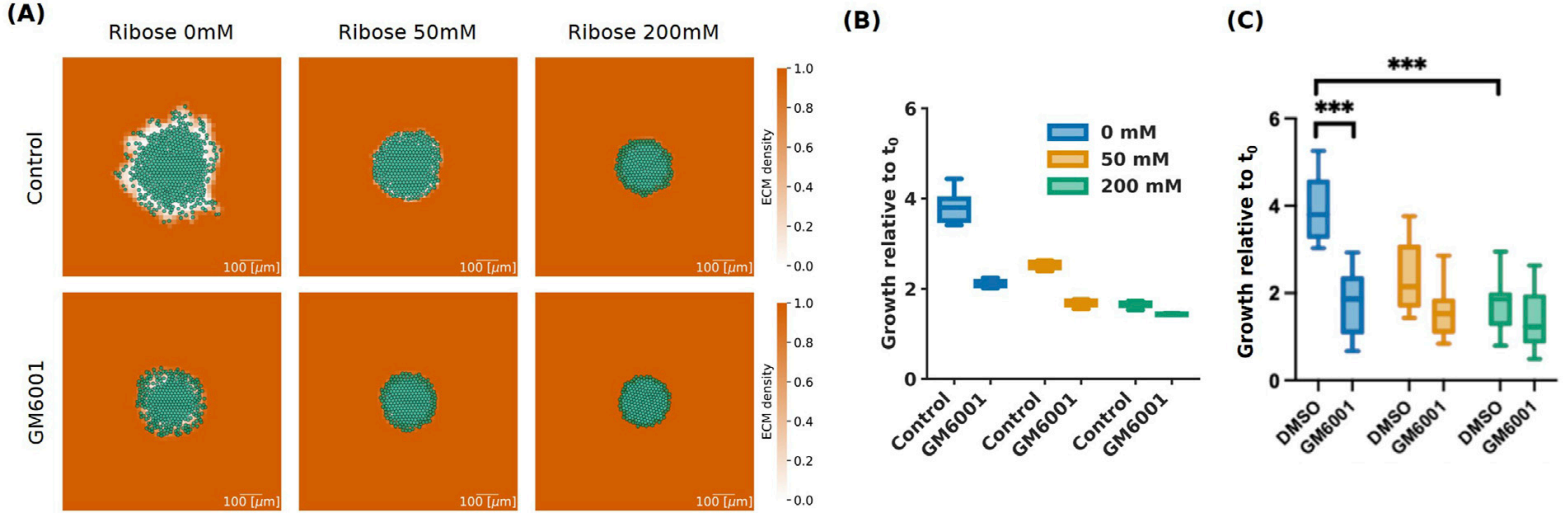
Simulations for invasive (HCC 1954) cells with pan-MMP inhibitor GM6001 at 72 h. **(A)** Simulation images of invasive cells with high (Control) and low (GM6001) degradation rates, with ribose concentrations of 0 mM, 50 mM and 200 mM, at 72 h. The cells are represented in semi-transparent green and the ECM density is in orange taking values between 0 and 1 as shown in the colour bar. The scale bar on the bottom right of length 100 μ m. **(B)** Box plots of spheroid growth relative to the initial time $t_{0}$ of invasive cells with high (Control) and low (GM6001) degradation rates at 72 h. The ribose concentrations are represented in blue for 0 mM, orange for 50 mM and green for 200 mM. Mean and 25th/75th percentile with min/max are shown. **(C)** Adapted with permission from Jahin et al. (2023). Box plots of growth relative to the initial time $t_{0}$ of invasive cells (HCC 1954) at 72 h. Comparison between control condition (DMSO) and pan-MMP inhibitor treatment (GM6001) at ribose concentrations 0 mM (blue), 50 mM (orange) and 200 mM (green). Mean and 25th/75th percentile with min/max are shown. Asterisks indicate statistical significance following ANOVA testing with Sidak’s *post hoc* test (*** = p< 0.001).

Cancer cells remodel the extracellular matrix mechanically and proteolytically when invading, creating paths that facilitate the migration of nearby attached cancer cells (Walker et al., 2018). The cells mechanically apply forces to the ECM fibres by pushing or pulling the fibres when adhering to ligand binding sites, resulting in fibre displacement and orientation changes. Fibre orientation can direct migration, and the realignment of the collagen fibres has been associated with higher invasion (Provenzano et al., 2006). On the other hand, proteolytic remodelling involves enzymatic degradation of ECM fibres through the activity of matrix metalloproteinases (MMPs). It has been shown that ECM degradation by MMPs significantly contributes to cell invasion as it facilitates migration and realignment of the fibres (Itoh, 2015). Jahin et al. (2023) investigated the role of MMPs in fibre alignment and invasion by treating the invasive HCC1954 cells with the pan-MMP inhibitor GM6001. They observed that MMPs inhibition significantly reduces invasion for ribose 0 mM, but not for ribose 50 mM and 200 mM (Figure 6C).

In our model, ECM remodelling is represented by the degradation rate parameter $r_{\mathrm{deg,0}}$ , which is responsible for changes in ECM density. We compared the experimental results in control conditions (DMSO) with the invasive cells simulations discussed in Section 3.2. Following the parameter analysis from Section 3.1 (Supplementary Figure S3), we choose the degradation rate matching the experimental results of growth relative to the initial time after 72 h of the invasive HCC1954 cell line with the addition of the pan-MMP inhibitor GM6001 (Figure 6C). We selected the degradation rate $r_{\mathrm{deg,0}}=$ 0.0004 ${\text{min}}^{-1}$ . This non-zero value arises because the pan-MMP inhibitor only blocks ECM degradation, but not mechanical remodelling of the fibres, which makes it possible for the cells to locally change the density of the ECM even without degrading the fibres. Simulation images at 72 h for invasive cells with high (Control) and low (GM6001) degradation rates are shown in Figure 6A.

Similarly to the results from the experimental data by Jahin et al. (2023) shown in Figure 6C, spheroid area growth relative to the initial time after 72 h was significantly reduced for ribose 0 mM going from ∼ 4 in the control conditions simulation (Control) to ∼ 2 with the reduced degradation rate (GM6001) (Figure 6B). At ribose concentration of 50 mM spheroid growth was less affected by the MMP inhibition, both in *vitro* and in the simulations. Finally, at 200 mM, both the Control and the reduced degradation rate (GM6001) conditions have similar spheroid sizes in the simulations after 72 h, which is in line with the experimental data in the DMSO and GM6001 conditions (Figure 6C).

## 4 Discussions

In this paper, we presented a hybrid discrete-continuous model built in PhysiCell (version 1.12.0) to describe the interactions between cells modelled as discrete agents and the extracellular matrix as a continuum. Our model incorporates cell-cell and cell-ECM adhesion and repulsion, ECM remodelling, and cell proliferation with associated inhibition of proliferation function, allowing us to investigate the critical role of cell-ECM interactions in cancer spheroids.

Our findings indicate that increased cell-ECM adhesion promotes invasion, while ECM degradation significantly influences spheroid growth. Notably, we observed a non-monotonic effect of ECM degradation: increasing degradation enhances growth due to reduced matrix confinement, yet excessive degradation limits migration for the cells at the edge of the spheroid, ultimately restricting invasion. The cell’s maximum speed is reached when the ECM density ρ is equal to 0.5 (Equation 7). Therefore, when the matrix is degraded too quickly, the ECM density quickly reaches values below 0.5, making the cells migrate slower. Typically cells do not over-degrade the ECM as the relationship between adhesion and cell survival is crucial. Anoikis, a programmed cell death mechanism in anchorage-dependent cells, highlights the necessity for ECM attachments since the communication between proximal cells and between cells and ECM provide essential signals for growth or survival (Kim et al., 2012). However, it has been found that cancer cells undergoing EMT can acquire anoikis resistance (Kim et al., 2012). In our model, we assume that the target value for ECM degradation is zero density (Equation 1). This can potentially be a limitation of our model leading to wrong predictions for high degradation rates. We also observed that the Delaunay mean distance for invasive cells at ribose concentration 0 mM does not monotonically increase, which contrasts with the interpretation we could derive from the results depicted in Figure 5Fiii. As shown in Supplementary Figure S4Biii, we found that the Delaunay mean distance reaches its peak between 24 and 48 h before gradually decreasing. This unexpected result could be an artifact of how we defined ECM remodelling in our model. However, this prediction could be validated experimentally by measuring the Delaunay mean distance across a greater number of time points. Finally, we found that lower rates of ECM degradation and migration speeds result in more symmetrical and compact spheroids. In contrast, higher degradation and migration speeds lead to increased cell detachment and protrusion formation at the spheroid’s edge.

We replicated the experiments carried out by Jahin et al. (2023) that investigated the impact of ribose-induced collagen stiffening on the invasion of two parental breast cancer cell lines: the non-invasive MCF7 and the invasive HCC 1954. We differentiated the cell lines based on their cell-ECM interaction parameters: ECM degradation rate $\left(r_{\mathrm{deg,rib}}\right)$ , which controls the ECM remodelling by the cells (Equation 1), and maximum cell-ECM interaction speed $\left(S_{\mathrm{rib}}\right)$ , which affects the cell’s movement (Equation 4). We assigned low cell-ECM interaction parameter values to the non-invasive cells and high cell-ECM interaction parameter values to the invasive cells. Assuming that higher ribose concentrations reduce ECM degradation and migration speed, our model successfully predicted a decrease in spheroid area growth with increasing ribose concentration, in line with the experimental observations of Jahin et al. (2023). Furthermore, we confirmed that inhibiting ECM degradation reduced spheroid growth in the invasive cell line.

In our current model, we represent the collagen fibre matrix as a homogeneous density and treat the ribose as a separate quantity that directly impacts ECM remodelling and cell migration. However, a more comprehensive framework of the matrix would benefit from incorporating additional ECM properties, such as fibre orientation, alignment and cross-linking (Metzcar et al., 2025; Noël et al., 2024). Fibre orientation and alignment affect the directed migration of cells, which is a process correlated with enhanced spheroid invasion. Furthermore, as cells dynamically remodel the ECM, fibre orientation and alignment change not only locally but also at greater distances. The addition of ribose increases cross-linking between fibres and impacts both the chemical and mechanical remodelling of the ECM by cancer cells. Furthermore, cancer cells also contribute to ECM deposition and cross-linking. Our modelling framework is adaptable and allows for the integration of these additional ECM properties, such as fibre orientation and alignment, as in Metzcar et al. (2025). In subsequent phases of the model development, we plan to implement these features and investigate their effects on spheroid growth.

The ECM can also be characterised by its stiffness, rather than by its density and ribose concentration (Poonja et al., 2023). However, our focus was on understanding how ribose-induced collagen stiffening specifically affected cancer spheroid growth and invasion. As previously mentioned, ECM stiffness can be modulated through various methods, each impacting different properties of the ECM and ultimately influencing the behaviour of the cells, thereby affecting the spheroid growth and invasion. It would be interesting to explore how these different stiffening methods could be reproduced in the current model. Additionally, it has been observed that the timing of ECM stiffening can either inhibit or promote cancer cell invasion, highlighting the complex relationship between ECM stiffness and cancer cell invasion (Staneva et al., 2018). Matrix stiffening after cancer invasion begins promotes further spheroid invasion, while a stiff matrix surrounding the spheroid at the early stages prevents invasion.

In addition to integrating more ECM features, future iterations of our model could incorporate diffusible nutrients and cell death mechanisms (Botte et al., 2023; Ruscone et al., 2023; Mancini et al., 2024; Macnamara et al., 2024). Currently, our model employs an inhibition of proliferation function based on the number of neighbouring cells and ECM density (Equation 8). While this approach provides a basic setup, a more comprehensive model would directly account for the effect of pressure, nutrient availability and cell death on proliferation dynamics. Another extension to the current model could involve incorporating various cell types, such as cancer-associated fibroblasts, which play a role in ECM remodelling by depositing ECM components (Jahin et al., 2023; Metzcar et al., 2025). However, incorporating these additional components into a hybrid model poses challenges, particularly with respect to parameter validation and mathematical function accuracy. This is worsened by the scarcity of relevant data. For instance, we lack the data necessary to distinguish between the proliferation and death of cancer cells within the spheroid.

Adding more features to the model would likely increase the variability of the results. Currently, the variability in the *in silico* model is considerably lower than that observed in the *in vitro* experiment. However, this is not necessarily problematic, as we are making a qualitative comparison based on mean values. The *in silico* model is a simplified description of the more complex *in vitro* model. For instance, in our simulations, we assume the ECM density to be initially homogeneous, contributing to the reduced variability of the results.

It is also important to note that our model operates within a constrained 3D space. Although we conceptually address the impacts of the ECM on cell migration in 3D, all simulations and analyses were restricted to 2D. We chose this approach to mimic the original 2D data: thin z-slices (microscopy images) of 3D spheroids. Additionally, the ECM framework developed by Metzcar et al. (2025) in PhysiCell currently supports only constrained 3D simulations, representing single slices of a 3D environment. This limits the model’s ability to capture the effects of the 3D environment and unconstrained cell movement on spheroid behaviour. Using the knowledge gained in this quickly executing constrained 3D model, we plan to model a fully 3D spheroid as future work.

In conclusion, the interactions between cancer cells and the extracellular matrix in 3D cancer spheroid growth are intricate and not yet fully understood. Our proposed model represents an initial attempt to account for the chemical and mechanical interactions within this context, paving the way for future research that integrates additional ECM properties and environmental factors.

## Data Availability

The datasets presented in this study can be found in online repositories. The names of the repository/repositories and accession number(s) can be found below: https://github.com/Margherita-Botticelli/PhysiCell-cancer-spheroid-ecm-stiffness.
