# Supplementary material for: A hybrid computational model of cancer spheroid growth with ribose-induced collagen stiffening
Source: Front Bioeng Biotechnol. 2025 Apr 9;13:1515962. doi: 10.3389/fbioe.2025.1515962 (PMC12014586; doi:10.3389/fbioe.2025.1515962)
Supplement: Supplementary file 1 [file DataSheet1.pdf]

# Supplementary Material

## 1 SUPPLEMENTARY TABLES

### Simulation parameters

| Symbol | Parameter | Values | Dimensions | Reference |
| --- | --- | --- | --- | --- |
| $\Delta t_{mech}$ | Mechanical time step | 0.1 | min | Ghaffarizadeh et al. (2018) |
| $\Delta t_{cell}$ | Phenotype time step | 6 | min | Ghaffarizadeh et al. (2018) |
| $\rho$ | ECM density | [0,1] | Dimensionless | Estimated |
| $rib$ | Ribose concentration | 0, 50, 200 | mM | Jahin et al. (2023) |
| $V$ | Maximum cell volume | 2494 | $\mu m^3$ | Ghaffarizadeh et al. (2018) |
| $R$ | Maximum cell radius | 8.4127 | $\mu m$ | Ghaffarizadeh et al. (2018) |
| $R_A$ | Maximum interaction radius | $1.25 \times R$ | $\mu m$ | Ghaffarizadeh et al. (2018) |
| $N_{max}$ | Neighbouring overcrowding threshold | 6 | Dimensionless | Metzcar et al. (2025) |
| $C_{cca}$ | Cell-cell adhesion strength | 0.4 | Dimensionless | Ghaffarizadeh et al. (2018) |
| $C_{ccr}$ | Cell-cell repulsion strength | 10 | Dimensionless | Ghaffarizadeh et al. (2018) |
| $T_{per}$ | Persistence time | 10 | min | Ghaffarizadeh et al. (2018) |

**Table S1.** List of parameters used in all simulations. All other parameters are set to their default values as used in PhysiCell 1.12.0 (Ghaffarizadeh et al., 2018).

### Parameter analysis

| Symbol | Parameter | Values | Dimensions | Reference |
| --- | --- | --- | --- | --- |
| $r_{div}$ | Proliferation rate | 0.0006, 0.0007, 0.0008 | $min^{-1}$ | Estimated |
| $S_0$ | Maximum cell-ECM interaction speed | (0, 1] | $\mu m \cdot min^{-1}$ | Estimated |
| $r_{deg,0}$ | ECM remodelling degradation rate | (0, 0.0256] | $min^{-1}$ | Estimated |
| $\sigma$ | Ribose effect strength on $S_0$ | [0, 0.045] | $mM^{-1}$ | Estimated |
| $\delta$ | Ribose effect strength on $r_{deg,0}$ | [0, 0.045] | $mM^{-1}$ | Estimated |

**Table S2.** List of parameters used in the parameter analysis in Section 3.1.

### Non-invasive cells

| Symbol | Parameter | Values | Dimensions | Reference |
| --- | --- | --- | --- | --- |
| $r_{div}$ | Proliferation rate | 0.00072 | $min^{-1}$ | Ghaffarizadeh et al. (2018) |
| $r_{deg,0}$ | ECM remodelling degradation rate | 0.0001 | $min^{-1}$ | Estimated |
| $S_0$ | Maximum cell-ECM interaction speed | 0.1 | $\mu m \cdot min^{-1}$ | Estimated |
| $\delta$ | Ribose effect strength on $r_{deg,0}$ | 0.02 | $mM^{-1}$ | Estimated |
| $\sigma$ | Ribose effect strength on $S_0$ | 0.035 | $mM^{-1}$ | Estimated |

**Table S3.** List of parameters used for the non-invasive cells in Section 3.2.

## Invasive cells

| Symbol | Parameter | Values | Dimensions | Reference |
|---|---|---|---|---|
| $r_{div}$ | Proliferation rate | 0.00072 | $\text{min}^{-1}$ | Ghaffarizadeh et al. (2018) |
| $r_{deg,0}$ | ECM remodelling degradation rate | 0.0032 | $\text{min}^{-1}$ | Estimated |
| $S_0$ | Maximum cell-ECM interaction speed | 0.7 | $\mu m\cdot$ $\text{min}^{-1}$ | Estimated |
| $\delta$ | Ribose effect strength on $r_{deg,0}$ | 0.02 | $\text{mM}^{-1}$ | Estimated |
| $\sigma$ | Ribose effect strength on $S_0$ | 0.035 | $\text{mM}^{-1}$ | Estimated |

**Table S4.** List of parameters used for the invasive cells in Sections 3.2 and 3.3.

## Invasive cells with pan-MMP inhibitor

| Symbol | Parameter | Values | Dimensions | Reference |
|---|---|---|---|---|
| $r_{div}$ | Proliferation rate | 0.00072 | $\text{min}^{-1}$ | Ghaffarizadeh et al. (2018) |
| $r_{deg,0}$ | ECM remodelling degradation rate | 0.0004 | $\text{min}^{-1}$ | Estimated |
| $S_0$ | Maximum cell-ECM interaction speed | 0.7 | $\mu m\cdot$ $\text{min}^{-1}$ | Estimated |
| $\delta$ | Ribose effect strength on $r_{deg,0}$ | 0.02 | $\text{mM}^{-1}$ | Estimated |
| $\sigma$ | Ribose effect strength on $S_0$ | 0.035 | $\text{mM}^{-1}$ | Estimated |

**Table S5.** List of parameters used for the invasive cells with pan-MMP inhibitor in Section 3.3.

# 2 SUPPLEMENTARY FIGURES

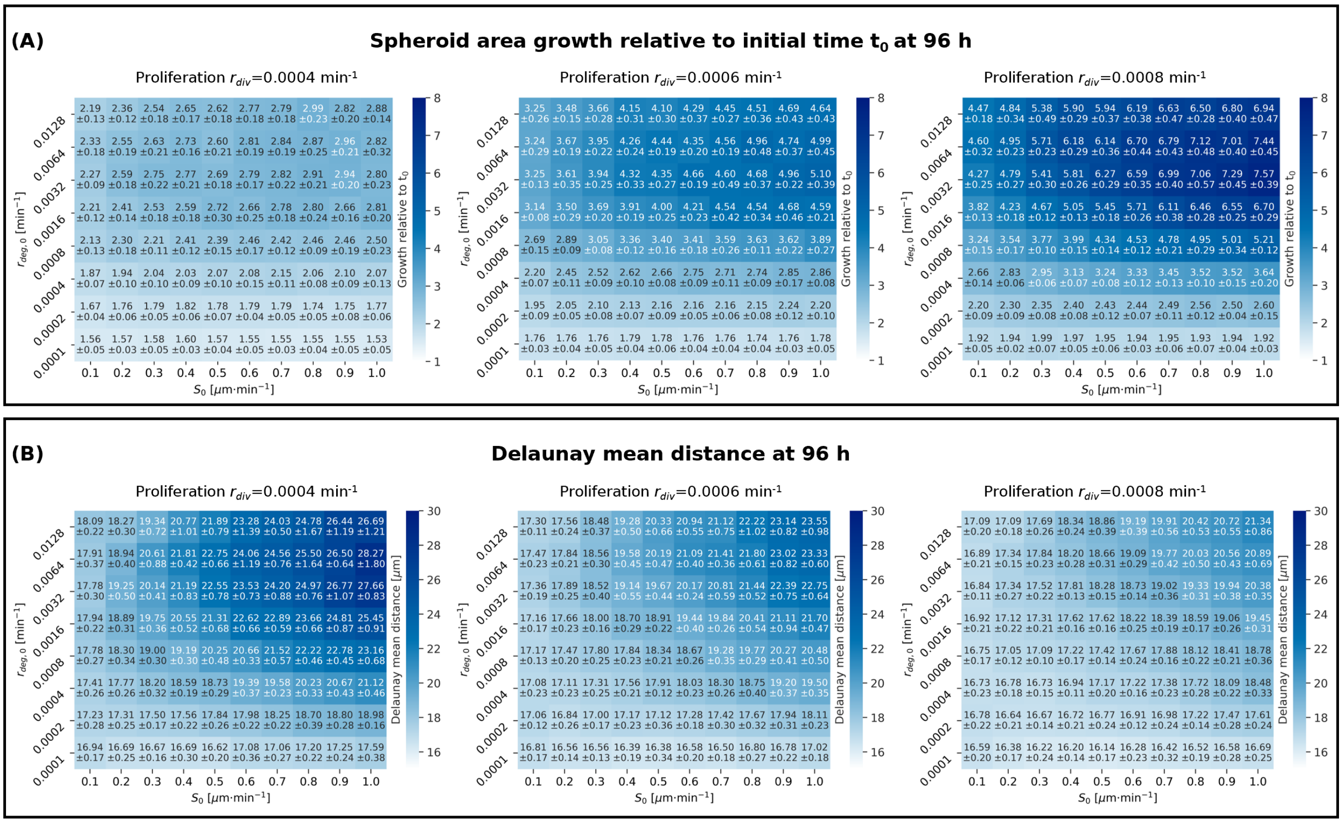


**Figure S1.** Heatmaps showing the effects on spheroid area growth relative to the initial time $t_0$ **(A)** and Delaunay mean distance **(B)** after 96 h with ribose concentration 0 mM of proliferation rate $r_{div}$ (columns), maximum cell-ECM interaction speed $S_0$ ($x$-axis) and degradation rate $r_{deg,0}$ ($y$-axis). Mean values and standard deviation over 10 replicates of the spheroid area growth relative to the initial time and Delaunay mean distance are shown. The colour intensity represents the mean values over 10 replicates of the spheroid area growth relative to the initial time ranging from 1 to 8 and Delaunay mean distance ranging from 10 $\mu$m to 30 $\mu$m, as shown in the colour bars. The text colour is white or black for contrast only.

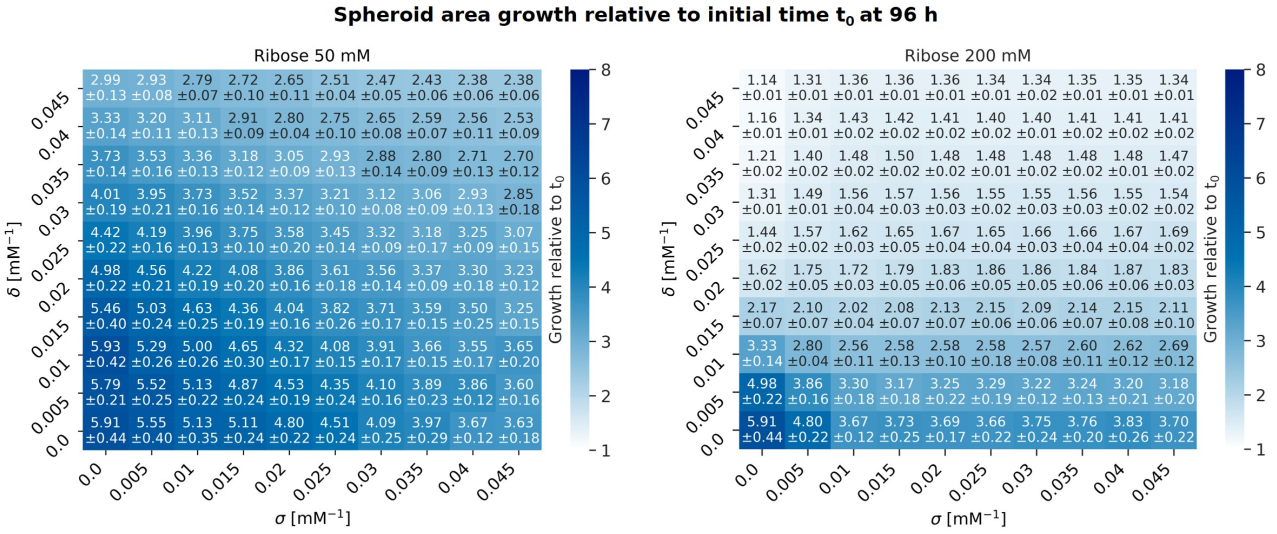


**Figure S2.** Heatmaps showing the effects of $\sigma$ ($x$-axis) and $\delta$ ($y$-axis) on spheroid area growth relative to the initial time $t_0$ after 96 h with ribose concentration 50 mM (left) and 200 mM (right), with proliferation rate $r_{div} = 0.00072$ min$^{-1}$. Mean values and standard deviation over 10 replicates of the spheroid area growth relative to the initial time are shown. The colour intensity represents the mean values of the spheroid area growth relative to the initial time ranging from 1 to 8, as shown in the colour bars. The text colour is white or black for contrast only.

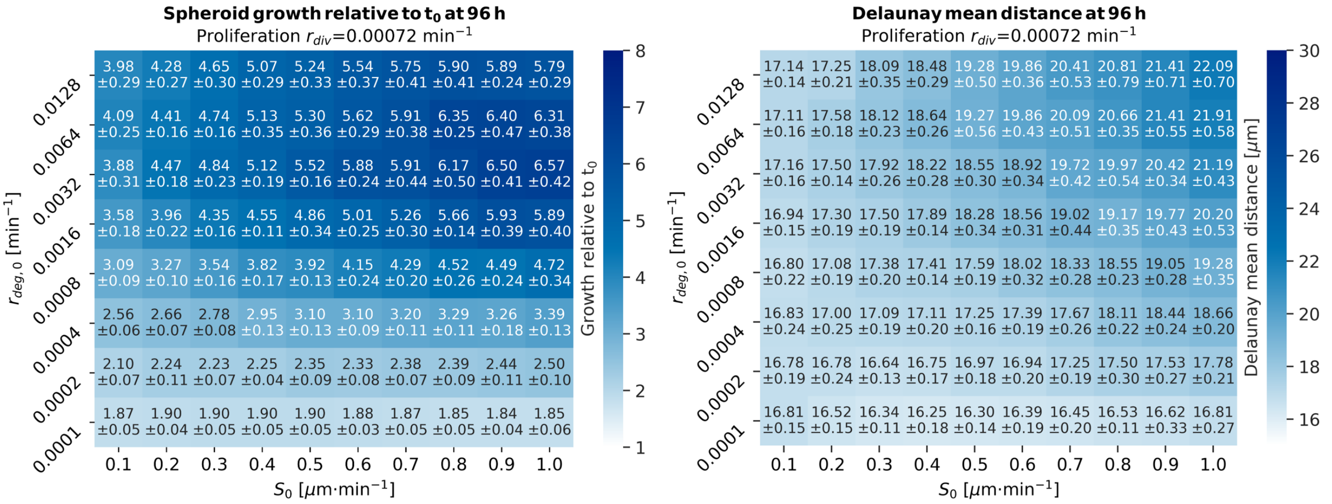


**Figure S3.** Heatmaps showing the effects of maximum cell-ECM interaction speed $S_0$ ($x$-axis) and degradation rate $r_{deg,0}$ ($y$-axis) on spheroid area growth relative to the initial time $t_0$ (left) and Delaunay mean distance (right) after 96 h with ribose concentration 0 mM and proliferation rate $r_{div} = 0.00072$ min$^{-1}$. Mean values and standard deviation over 10 replicates of the spheroid area growth relative to the initial time and Delaunay mean distance are shown. The colour intensity represents the mean values of the spheroid area growth relative to the initial time ranging from 1 to 8 and Delaunay mean distance ranging from 10 $\mu$m to 30 $\mu$m, as shown in the colour bars. The text colour is white or black for contrast only.

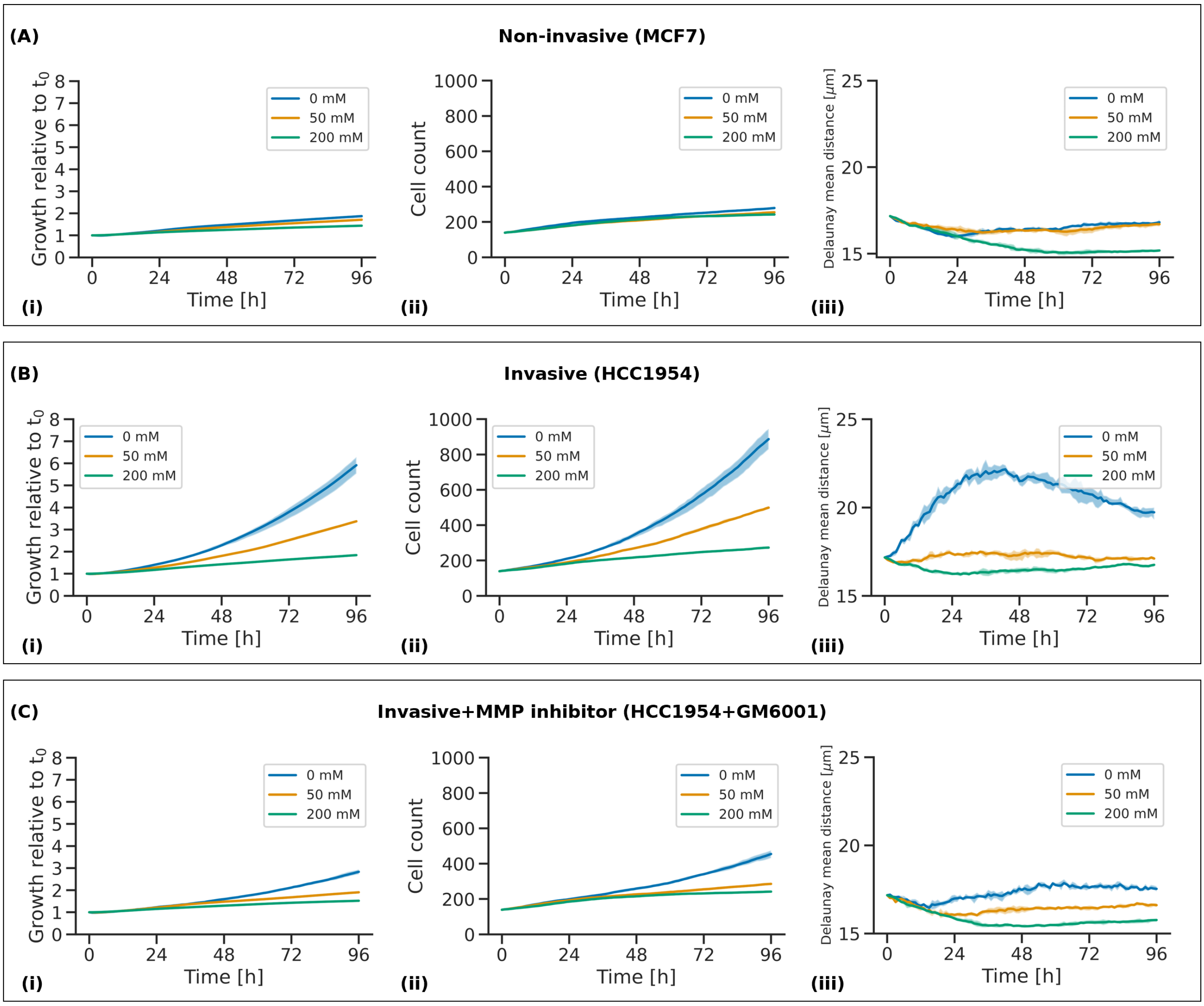


**Figure S4.** Results of simulations for non-invasive (MCF7) cells **(A)**, invasive (HCC1954) cells **(B)**, and invasive cells with the addition of MMP inhibition (HCC1954+GM6001) **(C)**) with ribose concentrations of 0 mM, 50 mM and 200 mM. Line plots of change over time of spheroid growth relative to the initial time $t_0$ **(i)**, cell count **(ii)** and Delaunay mean distance **(iii)** are shown. The ribose concentrations are represented in blue for 0 mM, orange for 50 mM and green for 200 mM. Mean and 25th/75th percentile over 10 replicates are shown.

## REFERENCES

Ghaffarizadeh, A., Heiland, R., Friedman, S. H., Mumenthaler, S. M., and Macklin, P. (2018). PhysiCell: an open source physics-based cell simulator for 3-D multicellular systems. *PLoS Comput. Biol.*, 14:e1005991.

Jahin, I., Phillips, T., Marcotti, S., Gorey, M.-A., Cox, S., and Parsons, M. (2023). Extracellular matrix stiffness activates mechanosensitive signals but limits breast cancer cell spheroid proliferation and invasion. *Frontiers in Cell and Developmental Biology*, 11:1292775.

Metzcar, J., Duggan, B. S., Fischer, B., Murphy, M., Heiland, R., and Macklin, P. (2025). A Simple Framework for Agent-Based Modeling with Extracellular Matrix. *Bulletin of Mathematical Biology*, 87(3):43.
